# A True Bug with a True but Unique Chela in 100 Million-Year-Old Amber †

**DOI:** 10.3390/insects17040431

**Published:** 2026-04-17

**Authors:** Carolin Haug, Fenja I. Haug, Marie K. Hörnig, Florian Braig, Joachim T. Haug

**Affiliations:** 1 Biocenter, Ludwig-Maximilians-Universität München, Großhaderner Straße 2, 82152 Planegg-Martinsried, Germanyjoachim.haug@palaeo-evo-devo.info (J.T.H.); 2 GeoBio-Center at LMU, Richard-Wagner-Straße 10, 80333 Munich, Germany; 3 University Medical Center Rostock, Medical Biology and Electron Microscopy Center, Strempelstr. 14, 18057 Rostock, Germany; marie.hoernig@palaeo-evo-devo.info; 4 Ecology and Genetics Research Unit, University of Oulu, Pentti Kaiteran katu 1, 90570 Oulu, Finland; florian.braig@palaeo-evo-devo.info

**Keywords:** Kachin amber, Burmese amber, Cretaceous, Nepomorpha, Gelastocoridae

## Abstract

Grasping appendages in representatives of the group Insecta are usually of the sub-chelate (jackknifing) type. Only rarely do chelate appendages occur: *Carcinothrips* (Thysanoptera), Dryinidae (Hymenoptera), and Carcinocorini (Heteroptera). Here, we report a new find from about 100 million-year-old Kachin amber, representing, to our knowledge, the first case of a fossil of the group Insecta with a chelate type of appendage. This find is only the fourth case of an independent or convergent evolution of such a structure. A quantitative morphological comparison of over 2000 grasping structures reveals that the proximal part of the chela (in this case, the femur) has a unique shape in the fossil, unparalleled in the fossil and modern fauna. Moreover, some other new fossils reported do not possess such a shape. The mouthparts, forming a distinct beak, allow us to identify the new fossil with the distinct chela as a representative of Heteroptera. Its short beak, together with some other characteristics, indicates that it most likely is a representative of Nepomorpha. Many other details of the fossil are not well preserved, but the overall appearance shows similarities to extant and fossil representatives of Gelastocoridae, a group of terrestrial predators.

## Simple Summary

In insects, grasping legs are usually like jackknives, with one part moving back against another part. Only few insects have legs working like a forceps (called chelate appendages); such legs occur in some species of thrips (Thysanoptera), solitary wasps (Hymenoptera), and true bugs (Heteroptera). In this study, we present a new insect preserved in amber from Kachin, Myanmar (ca. 100 million years old). This find appears to represent the first fossil insect with a forceps-like leg. It is only the fourth time that such a structure evolved independently in an insect. We compared the shapes of over 2000 grasping structures. We found out that in the forceps-like leg of the new fossil, the part closer to the body has a shape unknown from any other fossil or modern representative. The shape is also different in some other newly reported fossils. On the head, the new fossil with the unique forceps-like leg possesses a distinct short beak. This characteristic, together with some others, allows us to identify it as a true bug, most likely a true water bug (Nepomorpha). In many other aspects the fossil is not well preserved but, overall, it looks similar to toad bugs (Gelastocoridae), which are terrestrial predators.

## 1. Introduction

**Figure 1:**
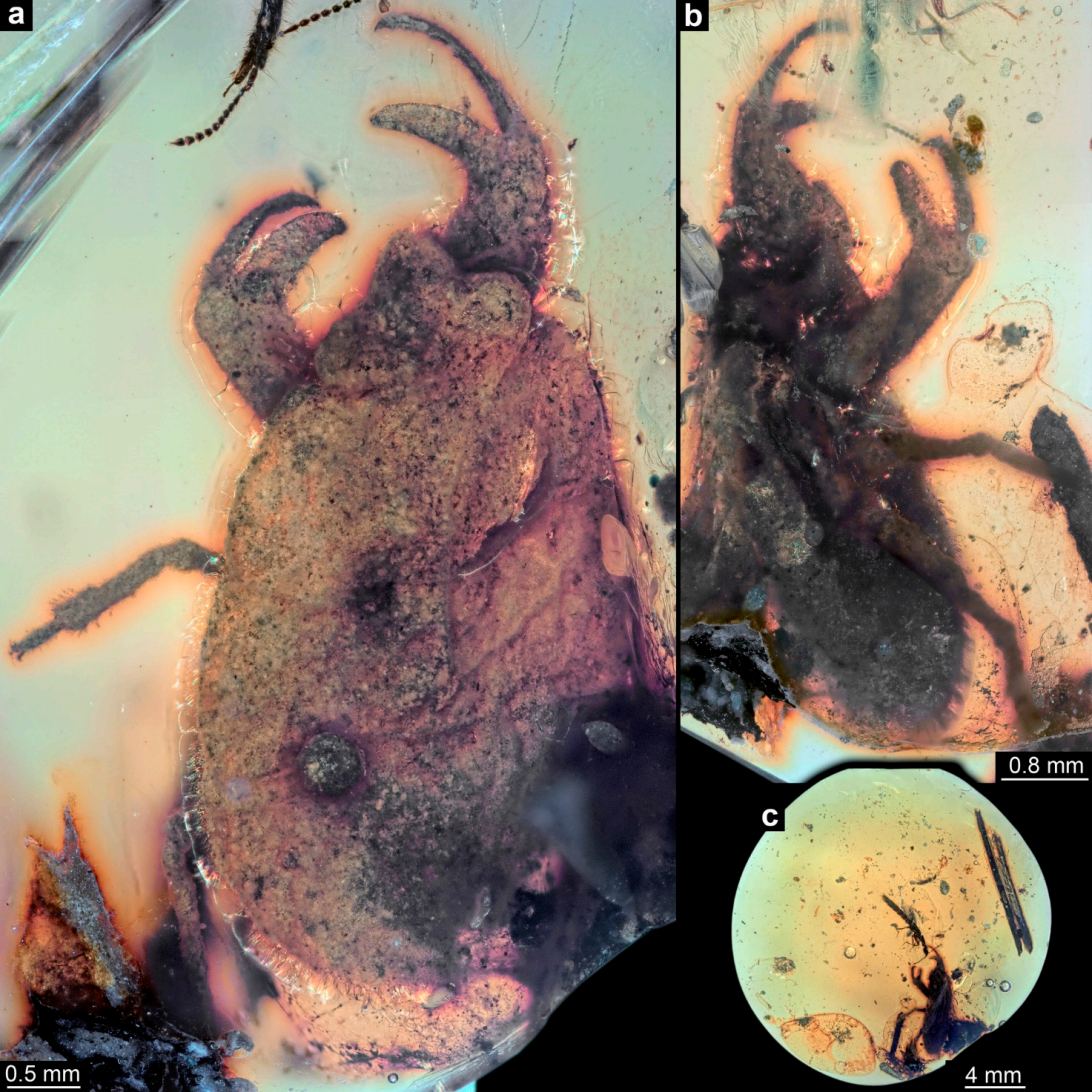
Holotype of *Carcinonepa libererrantes* n. gen. n. sp., PED 4665, Kachin amber (Myanmar). (**a**). Dorsal view. (**b**). Latero-ventral view. (**c**). Entire amber piece with syninclusions (see details in Supplementary Figure S1).

Grasping appendages have evolved repeatedly within the group Insecta for various purposes, most impressively in the use of catching prey, as is well known in praying mantises, mantis lacewings, or raptorial true bugs. Most of these appendages follow the same basic principle, namely that of a sub-chelate arrangement, often described as jackknife arrangement, in which a distal part (usually the tibia, or tibia and tarsus together) folds backwards against a more proximal part (usually the femur) (e.g., [1] figure 1 p. 139). This arrangement is different from grasping appendage arrangements that we know from closer relatives such as crabs and lobsters. In the latter, the proximal part has a distal protrusion forming a fixed finger, making the distal part a movable finger; this arrangement is usually termed chelate, with the entire structure termed a chela ([1] figure 1 p. 139). While there are mantis lacewings with large protruding spines on the proximal part reminding of fixed fingers (e.g., [2] figure 5 p. 20), these are more proximal than in the chelae of crabs, making the distal finger still fold backwards, and they are therefore considered sub-chelate. Therefore, not only the presence or absence of a fixed finger seems usually sufficient to distinguish between a chelate or sub-chelate arrangement, but also the angle of closure seems to be considered. A third, somewhat intermediate, condition is sometimes referred to as pseudo-chelate (e.g., [3] figure 22 p. 91; [4] figure 4 p. 306), but the term has also been used quite differently (e.g., [5] figure 7A p. 229; [6] figure 71-11.5 p. 594; [7] figure 4 p. 213). In such a structure, there is often a short fixed finger, but the face against which the distal movable finger closes is perpendicular to the main axis of the appendage. For closing angles smaller than 90°, appendages seem to be termed chelate; for those larger than 90°, they appear to be usually termed sub-chelate.

**Figure 2:**
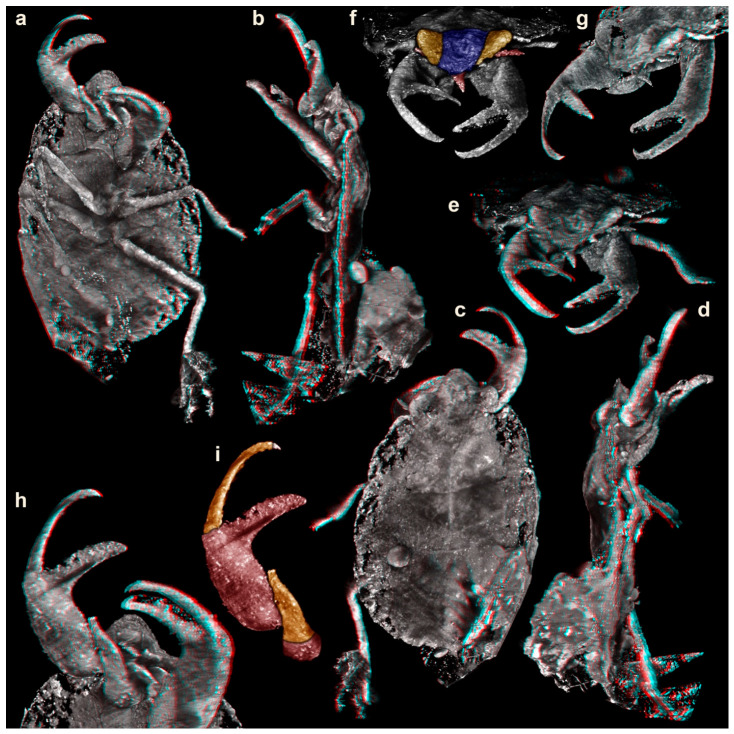
Volume renderings based on µCT-data of the holotype of *Carcinonepa libererrantes* n. gen. n. sp., Kachin amber (Myanmar), PED 4665, from different views. (**a**–**e**,**g**,**h**) are stereo anaglyphs; please use red–cyan glasses to view them. (**a**). Ventral view. (**b**). Lateral view from left side. (**c**). Dorsal view. (**d**). Lateral view from right side. (**e**). Anterior view. (**f**). Anterior view with head capsule (blue), eyes (yellow), beak and antenna (red) color-marked. (**g**). Antero-lateral view. (**h**). Close-up on chelae. (**i**). Chela color-marked.

**Figure 3:**
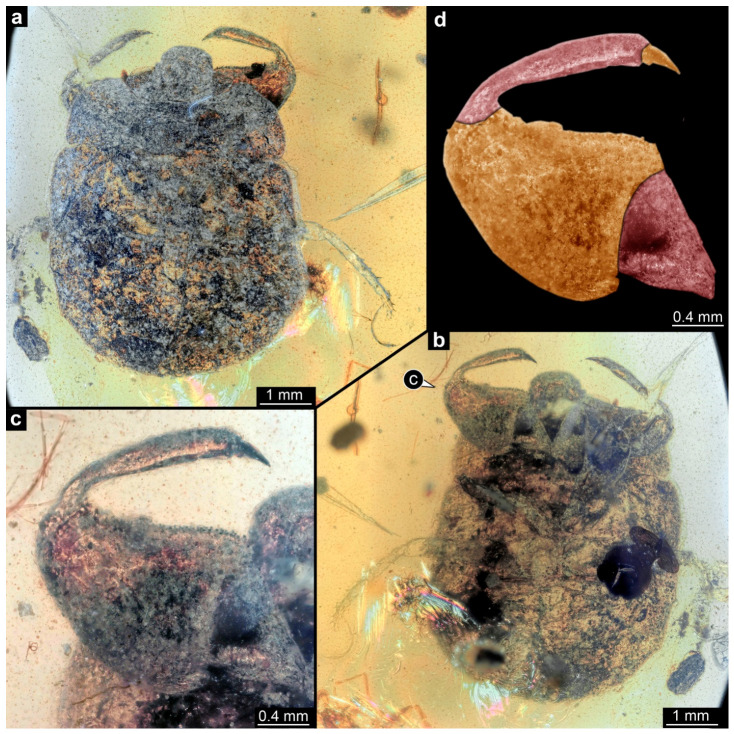
True bug, most likely a representative of the group Gelastocoridae, PED 4666, Kachin amber (Myanmar). (**a**). Dorsal view. (**b**). Ventral view. (**c**). Close-up of one foreleg in ventral view. (**d**). Color-marked version of (**c**).

**Figure 4:**
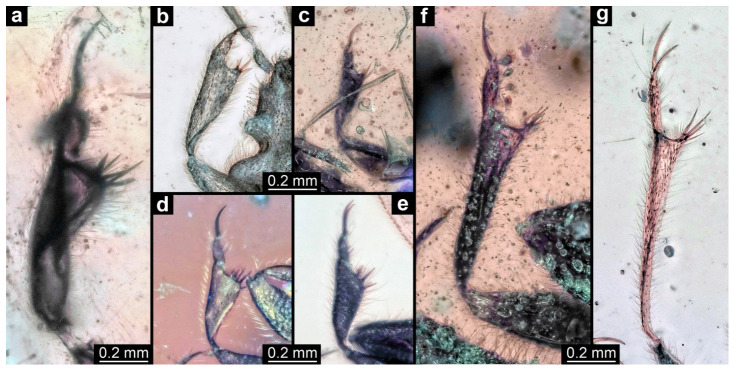
Forelegs of different representatives of Enicocephalomorpha. (**a**). PED 2110. (**b**). PED 3569. (**c**). PED 2208. (**d**). PED 4002. (**e**). PED 2470. (**f**). PED 2882. (**g**). PED 2502.

**Figure 5:**
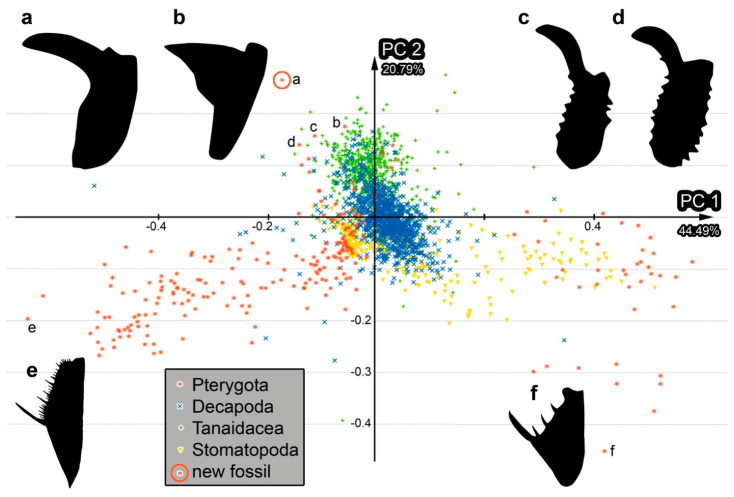
Scatterplot of PC2 vs. PC1 values of the shape analysis. The small letters indicate the position of certain specimens in the scatterplot, and their shapes are additionally depicted. (**a**). *Carcinonepa libererrantes* n. gen. n. sp., PED 4665. (**b**). Extant representative of Gelastocoridae, ChIN_0077. (**c**,**d**). Extant representatives of Carcinocorini. (**c**). ChIN_0006. (**d**). ChIN_0007. (**e**). Extant mantis lacewing, NMA0350. (**f**). Extant praying mantis, MaFe_048. For details on the specimens, see Supplementary Table S1.

Most raptorial appendages in Insecta seem to represent the case of a sub-chela, but there are a few examples of pseudo-chelate and even chelate ones. Generally, three lineages have been suggested to have evolved chelate appendages: ingroups of Thysanoptera (thrips), namely *Carcinothrips* (only females) ([8] figure 1 p. 11, figure 3 p. 12, figure 4 p. 13); Hymenoptera (wasps), namely Dryinidae (only females) ([9] figure 6 p. 552; [10] figure 2 p. 45; [11] figures 2, 3 p. 5; [12] figure 1 p. 275); and Heteroptera (true bugs), namely Carcinocorini ([1] figure 5 p. 145; [13] figures 2–4 pp. 797–798). The attachment structures of some lice (Phthiraptera) may resemble chelae at first glance, but a closer look reveals that these are in fact more complex. Here, the tibia has a short and stout finger-like protrusion against which the tarsus closes, but, in addition, the movable tarsal claw closes backwards against the tarsus [14]. A similar kind of double closure system is also known in peracaridan crustaceans and is termed carpo-chelate (e.g., [15] figure 1 p. 63; [16] figure 1 p. 57).

Here, we report a new fossil preserved in about 100 million-year-old Kachin amber, that has the clear characteristics of a true bug (Heteroptera) and possesses prominent chelate appendages. To our knowledge, this is the first report of a fossil representative of the group Insecta with a prominent true chela. It also represents (possibly) only the fourth case of evolution of such a true chela in Insecta.

## 2. Materials and Methods

### 2.1. Materials

In total, nine fossil specimens of true bugs (Heteroptera) were directly investigated. These specimens are deposited in the Palaeo-Evo-Devo Research Group Collection of Arthropods, Ludwig-Maximilians-Universität München (LMU Munich), Germany, under repository numbers PED 2110, 2208, 2470, 2502, 2882, 3569, 4002, 4665, and 4666. All these are preserved in about 100 million-year-old Kachin amber from Myanmar. Specimens were legally purchased on the trading platform ebay.com from three traders (burmitefossil, burmite-miner, monty-and-turner).

Comparative material of fossil and extant crustaceans (including representatives of Insecta; for systematics, see, e.g., [17,18]) with chelate and sub-chelate appendages was retrieved from the literature or from specimen collections, in total 2099 (Supplementary Tables S1 and S2). These include:

- Representatives of Insecta with chelate appendages;
- Representatives of Insecta with a large variety of sub-chelate appendages;
- Representatives of Malacostraca with chelate appendages;
- Representatives of Malacostraca with prominent raptorial sub-chelate appendages.

The investigated groups were:

- True bugs, i.e., representatives of the group Heteroptera (especially of the ingroups Nepomorpha, Phymatinae, and Enicocephalomorpha);
- True lice, i.e., representatives of the group Phthiraptera;
- Thrips, i.e., representatives of the group Thysanoptera;
- Praying mantises, i.e., representatives of the group Mantodea;
- Mantis lacewings, i.e., representatives of the group Mantispidae;
- Wasps, i.e., representatives of the group Dryinidae;
- Extant lobsters, crayfish and their relatives;
- Fossil relatives of modern lobsters;
- True crabs and false crabs, i.e., representatives of the group Meiura;
- Mantis shrimps, i.e., representatives of the group Stomatopoda;
- Representatives of the group Tanaidacea.

Specimens from natural history collections (Supplementary Table S1) originated from the Zoological State Collection Munich (ZSM), the Natural History Museum Berlin (MB), the Zoological Museum Copenhagen (ZMUC-CRU), the Senckenberg Naturmuseum Frankfurt (SMF), and the Muséum national d’histoire naturelle Paris (MNHN). The sample MNHN-IU (internal #3–#191) was collected on the coast of Congo, near Pointe-Noire (4°57′ S–11°25′ E, R.P.N. 21. GS. 2. Fds 100 m. 25 m W.O. 11/1/64. 20h00–20h15).

### 2.2. Documentation Methods and Image Processing

The nine directly investigated specimens were mounted in a petri dish with modeling clay. Glycerol was dropped onto the specimens; some specimens had to be completely immersed in glycerol. Then a cover slip was placed on the area of interest of the specimens. Photos of the animals were recorded on a Keyence VHX-6000 digital microscope (Keyence, Osaka, Japan) with a white background and a ring light. All images were recorded as composite images (stacks, panorama) and with HDR [19]. Adobe Photoshop CS2 and CS3 (Adobe, San José, CA, USA) were used to further optimize the resulting images.

For µCT, an Xradia 410 Versa-X-ray microscope (Zeiss, Oberkochen, Germany) was used at the Institut für Biowissenschaften, Lehrstuhl Allgemeine & Spezielle Zoologie, Universität Rostock. Scans were performed with the following settings: 4× objective, 40 kV, 8 W, 3 s exposure time, binning 2, pixel size = 4.621 µm. Two scans were vertically stitched. The visualization of the CT data was performed in Osirix (open access). Images were projected as volume renderings and as stereo images. The resulting red–blue stereo images were transformed into red–cyan stereo images in Adobe Photoshop CS3.

### 2.3. Shape Analysis

In total, 2107 specimens were included in the shape analysis (one of the directly investigated fossil specimens was not included in the analysis, as it was partly distorted). The proximal part of each grasping structure was outlined with a vector graphics program (Adobe Illustrator CS2, Adobe, San José, CA, USA) and used as a basis for the analysis. For representatives of Mantodea, Mantispidae, and some true bugs, this was the femur of the foreleg (endopod element 2 of post-ocular appendage 6). For lobsters, crayfish, and their relatives, as well as most crabs, this was the propodus of thoracopod 4 (endopod element 4 of post-ocular appendage 9). For some carrier crabs, it was the propodus of thoracopod 8 (endopod element 4 of post-ocular appendage 13). For representatives of Stomatopoda, this was the carpopropodus of thoracopod 2 or maxilliped 2 (a compound of endopod elements 3 and 4 of post-ocular appendage 7). For representatives of Tanaidacea, this was the propodus of thoracopod 2 (endopod element 4 of post-ocular appendage 7). For some true bugs, it was the tibia of the foreleg (endopod element 3 of post-ocular appendage 6). For representatives of Dryinidae, it was tarsus element 5 of the foreleg (part of endopod element 4 of post-ocular appendage 6).

We performed an elliptic Fourier analysis (EFA) with the free software SHAPE (ver. 1.3, open access) [20]; details in e.g., [2]. Hereby, a complex two-dimensional shape is decomposed to quantify its geometrical information, resulting in a harmonic sum of trigonometric functions weighted by harmonic coefficients describing the shape. This object can then be analyzed with multivariate tools [21,22]. We chose 20 harmonics to describe the variation of the dataset and aligned them on the basis of the first harmonic. The results were analyzed using principal component analysis (PCA) to reduce dimensionality and highlight the dimensions of largest variation. Plotting of the results was performed in OpenOffice (open access).

## 3. Results

### 3.1. Description of PED 4665

Specimen of interest in an amber piece with a large number of syninclusions, including mites, a pseudoscorpion, two beetles, and some collembolans (Figure 1c; Supplementary Figure S1). Body organized into head and trunk (Figure 1a,b and Figure 2a–d). Head triangular in dorsal view, laterally with bulging compound eyes (Figure 1a and Figure 2e–g). Close to the compound eyes, short laterally projecting appendages (antennae) apparent (Figure 2e,f). Mouthparts forming a short and cone-shaped beak-like structure (Figure 2e,f).

Trunk dorsally with three distinct subdivisions (Figure 1a and Figure 2c) interpreted as tergite of thorax segment 1 (pronotum), non-differentiable tergites of thorax segments 2 and 3 (meso- and metanotum; possibly separated by a faint V-shaped line) and non-differentiable remaining trunk (abdomen). Ventrally with three pairs of trunk appendages (legs; Figure 2a). Appendages of thorax segment 1 each with pronounced chela (Figure 2a,h,i). Proximal part of chela (femur) with a slightly curved fixed finger, medially pointing to about 90° against the main appendage axis. Distal part of chela (movable finger) composed of one very long element (tibia) and one very short element (tarsus); more slender than the fixed finger, curved to a similar degree as the fixed finger. Appendages of thorax segments 2 and 3 subsimilar, walking-type appendages.

### 3.2. Description of PED 4666

Body organized into head and trunk (Figure 3a,b). Head rounded triangular in dorsal view, laterally with bulging compound eyes (Figure 3a,b). Head appendages not distinguishable.

Trunk dorsally with four distinct subdivisions (Figure 3a), interpreted as the tergites of the thorax segments 1–3 (pro, meso-, metanotum) and non-differentiable remaining trunk (abdomen). Mesonotum with laterally developing wings. Ventrally with three pairs of trunk appendages (legs; Figure 3b). Appendages of thorax segment 1 each with prominent sub-chela (Figure 3c,d). Proximal part of sub-chela (femur) broad triangular. Distal part of sub-chela slender and slightly curved, composed of one very long element (tibia) and one very short element (tarsus) Appendages of thorax segments 2 and 3 subsimilar, walking-type appendages.

### 3.3. Short Description of the Grasping Structures of Other Directly Investigated Specimens

Of specimens PED 2110, 2208, 2470, 2502, 2882, 3569, and 4002, only the grasping structures are described for comparative aspects (Figure 4). In all specimens, the proximal part of the grasping structure is formed by the tibia; the distal part by the tarsus. The tibia is armed with several spines pointing medio-distally; its distal occlusion surface varies between about orthogonal to the main appendage axis (Figure 4g) and being inclined backwards (Figure 4e). The tarsus appears to consist of a single elongated proximal element and two distal claws. In the larger specimens, the tibia and the proximal part of the tarsus are rather elongated (Figure 4a,f,g), while they are stouter in the smaller specimens (Figure 4b–e).

### 3.4. Results of the Shape Analysis

The shape analysis resulted in six effective principal components (PCs) describing 90.5% of the overall variation in the dataset (details in Supplementary Files S1–S6). PC1 and PC2 together describe 65.3% of the overall variation. PC1 describes 44.5% and is dominated by the distinctness of the separation between the distal and proximal areas of the proximal part of the chela/sub-chela. PC2 explains 20.8% of the overall variation. It is dominated by the slenderness of the distal area of the proximal part of the chela/sub-chela.

## 4. Discussion

### 4.1. Identity of the Specimens PED 4665 and PED 4666

All directly investigated specimens in this study can be identified as representatives of the group Heteroptera. The most unusual specimen, PED 4665 (Figure 1 and Figure 2), is not entirely well preserved but still provides details of the mouthparts, which form a distinct but short conical beak. This morphology allows us to identify the specimen as a representative of Heteroptera with confidence. It is presumably a representative of Nepomorpha, which is supported by the short antennae and the possible absence of ocelli (Appendix 1 in [23]); the latter cannot be corroborated due to insufficient preservation quality. Further reaching identification is challenging as well, which prevents access to details by light microscopy or µCT.

Still, the overall appearance strongly resembles another specimen reported here, PED 4666 (Figure 3). Both share the prominent triangular head (due to the strongly developed compound eyes), prominent pronotum, and mesonotum, without distinct further subdivision of the posterior trunk. Specimen PED 4666 strongly resembles modern representatives of the group Gelastocoridae, an ingroup of Nepomorpha with shortened raptorial forelegs (Appendix 1 in [23]; [24] figures 1–5 p. 62; [25]). This resemblance makes it possible that PED 4665 is also a representative of the group Gelastocoridae. Unfortunately, further distinct characters of Gelastocoridae such as the composition of the antenna, the exact tarsal formula, or the surface texture of thorax and forewings (Appendix 1 in [23]) can neither be accessed in PED 4665 nor in PED 4666. PED 4665 is a bit more elongate, resembling, for example, representatives of Belostomatidae ([26] figure 2C p. 50). However, the shape of the coxae and tibiae of the hindlegs does not resemble those of Belostomatidae (Appendix 1 in [23]).

While the shape of the chela of specimen PED 4665 is special, as clearly demonstrated by the quantitative morphological analysis (Figure 5), the specimen plotting closest to it is an extant representative of Gelastocoridae. The structure of the proximal part of this appendage strongly resembles that of representatives of Gelastocoridae ([27] figure 1c p. 4), further supporting that the fossil PED 4665 could be a representative of this group. The syninclusions (Supplementary Figure S1), which are terrestrial elements, are congruent with a possible terrestrial lifestyle like in extant representatives of Gelastocoridae, while freshwater elements could have been seen as an argument for a different closer relationship within Nepomorpha.

Both specimens show a dorsal subdivision into only three distinct parts along the trunk, which likely represent the pronotum, mesonotum plus metanotum, and the abdomen. This subdivision can be seen in other fossils, but also in modern representatives of Nepomorpha ([26] figure 2C p. 50). This morphology indicates that both are immature. There is a slight indication of a V-shaped fold on the second trunk part on PED 4665 (Figure 1a and Figure 2c), which might indicate the line between the mesonotum and the metanotum. If this assumption is correct, the developing wings should already be of a certain size (otherwise the line would be straighter), indicating that PED 4665 might be at postembryonic stage 3–5 ([24] figures 2–4 p. 62). In PED 4666, the line between the mesonotum and the metanotum is more clearly visible, as are the developing wings (Figure 3a), indicating postembryonic stage 5 ([24] figure 4 p. 62).

### 4.2. Fossil Record of Gelastocoridae

So far, few fossils of Gelastocoridae have been reported, but these reports come from Kachin amber ([28]; [29] figure 3g,h p. 98). Personal observation reveals that fossils of this group are quite commonly offered on the market. The previously reported fossils have less distinct grasping appendages in the sense that the distal movable finger seems less “condensed” and has a pair of rather prominent tarsal claws ([28] figure 5 p. 42, figure 10 p. 43; [29] figure 3g p. 98). This morphology is more similar to that of modern representatives of Gelastocorinae ([27] figure 1c p. 4; although one of the fossils in [28] was interpreted as a representative of Nerthrinae). The two new fossils that might be representatives of Gelastocoridae also have derived movable fingers that appear to be more condensed, like many modern representatives of Nerthrinae ([25] figures 6, 7 p. 37). Still, the fossil PED 4665 shows a morphology so far unknown in any representative of the group in the proximal part of the grasping structure.

### 4.3. Taxonomic Treatment

**

Heteroptera Latreille, 1810

Nepomorpha Popov, 1968

?Gelastocoridae Kirkaldy, 1897

**

*Carcinonepa* gen. nov.

**

Etymology: “Carcino” as a reference to crabs due to the prominent chela, a common denomination referring to chelate appendages in Insecta as in *Carcinothrips* or *Carcinocoris*; “nepa” as a reference to the group Nepomorpha.

**

Type species: *Carcinonepa libererrantes*

**

Diagnosis: as for the species

**

*Carcinonepa libererrantes* sp. nov.

**

Etymology: “liberi” as the Latin word for “children”, “errantes” as the Latin word for “wandering”, the compound being the latinized version of the band name “Stray Kids”, a famous K-pop band and the favorite band of one of the authors (FIH); also for the pose of the holotype resembling the signature hand sign of Stray Kids.

**

Holotype: PED 4665, Palaeo-Evo-Devo Research Group Collection of Arthropods, Ludwig-Maximilians-Universität München (LMU Munich), Germany

**

Type locality: Hukawng Valley, Kachin State, Myanmar; Late Cretaceous

**

Diagnosis: Nepomorphan bug with pronounced true chelae on the forelegs. Proximal part of each chela with fixed finger formed by the femur; movable finger formed by the tibia and small tarsus.

### 4.4. Identity of the Other New True Bugs in Kachin Amber

All other specimens directly investigated in this study can be identified as representatives of Enicocephalomorpha, based on the shape of the front leg (Figure 4). There has been a species of Enicocephalomorpha formally described from Kachin amber [30], but its morphology does not resemble that of any of the specimens investigated herein. The newly reported specimens, all of them fully winged, and hence adults, show a strong variability in the overall shape of the front legs, with some of them being more elongate and others being stouter; also, the size difference is quite strongly expressed. These differences indicate that there are several species of Enicocephalomorpha in Kachin amber, which should be further explored in future studies.

### 4.5. Chelate Appendages in the Group Insecta

As noted, grasping appendages in Insecta are commonly sub-chelate. In such cases, either the tibia acts as the movable finger (e.g., Mantodea: [31]; some species of Mantispidae: [32]), or the tibia and the entire tarsus (many species of Nepomorpha: [27]; some species of Mantispidae: [33]), or the tibia and the proximal part of the tarsus (e.g., in some species of Mantispidae: [32]; Rhachiberothidae/Rhachiberothinae: [34]; see [35] for the taxonomic uncertainty) may act as the functional movable finger.

The few cases of truly chelate appendages differ from this arrangement. In the case of Dryinidae, the proximal part of the chela with the fixed finger is formed by tarsus element 5; the movable finger is formed by the tarsal claw (e.g., [9] figure 6 p. 552). In the case of Carcinocorini, the proximal part of the chela with the fixed finger is formed by the tibia; the movable finger is formed by the undivided tarsus ([1] figure 5 p. 145).

In the case of *Carcinothrips*, it is partly unclear whether the grasping appendage indeed represents a chela. The occlusion surface is positioned around 90° towards the main axis (in females; [8] figure 1 p. 11, figure 3 p. 12, figure 4 p. 13; males clearly have sub-chelate appendages; [8] figure 2 p. 12). This appendage may therefore be considered pseudo-chelate but could also be understood as chelate. The proximal part of the chela with the fixed finger is formed by the femur. The movable finger seems to be formed by the tibia, while the tarsus is present but is reduced in size and does not seem to contribute to the functional finger. Hence, the two structures involved are similar to the ones forming the sub-chela in Mantodea (see above).

Grasping appendages of Enicocephalomorpha may be considered pseudo-chelate, as the occlusion surface appears to be positioned around 90° towards the main axis ([36] figure 6 p. 222; [37] figure 4 p. 8). Especially in the fossils reported here, the tibiae, in addition, have short finger-like spines that make them functionally a bit more like chelae (Figure 4). The proximal part is the tibia; the distal finger is formed by an undivided tarsus and the separate paired tarsal claws.

*Carcinonepa libererrantes* is similar to its possible relatives, i.e., representatives of Gelastocoridae, in that the distal movable finger is composed of the tibia and a short tarsus (Figure 3; [25] figures 6, 7 p. 37). Furthermore, the proximal part is also the femur ([27] figure 1c p. 4), but unlike in (other?) representatives of Gelastocoridae, *Carcinonepa libererrantes* has a prominent fixed finger. The finger almost extends sideways, reminding one of a pseudo-chela at first glance. However, the prominence of the finger and the fact that it is slightly less than 90° off axis make this a clear chela. However, the specimen also reveals that measuring the exact closure angle can be a challenging task, if the fixed finger, as in the fossil, does not protrude in a straight way towards the median but additionally protrudes slightly anteriorly and then curves slightly posteriorly.

With this combination, the exact morphology of the chela of *Carcinonepa libererrantes* seems unique, as further emphasized by the fact that the shape of the femur is different from all other known proximal parts of chelae (Figure 5). Still, the morphology of at least one extant species of Gelastocoridae provides a kind of bridging type of morphology, with a triangular protrusion functionally acting as a sideways directed finger ([25] figure 13 p. 38), making this likely a type of pseudo-chela. These morphologies can be understood as being derived from a more sub-chelate morphology in other species of Gelastocoridae (e.g., [25] figures 6, 7 p. 37).

### 4.6. Convergent Evolution of Grasping Structures

In the few cases of truly chelate grasping structures in Insecta, the different substructures forming the chelae strongly indicate their independent evolution. In the present case, it seems that within Gelastocoridae, a chelate grasping structure evolved from a sub-chelate one. This route also appears to be the case for Carcinocorini within Phymatinae ([1] figure 5 p. 145). Other chelate appendages do not seem to have evolved via this route. Interestingly, the chelae of Gelastocoridae and of Phymatinae plot closely together in the morphospace (Figure 5). This similarity seems to concern the orientation of the fixed finger, which is more sideways in both cases of true bugs, while in common chelae, the fixed finger is more in line with the main axis of the appendage. While we could argue that this is a phylogenetic frame, it is more likely a truly functionally coupled one. In both cases, we see the same path, although different substructures are involved: the fixed finger arises from the tibia in Phymatinae ([1] figure 5 p. 145) but from the femur in *Carcinonepa libererrantes.* This suggests that if we find other cases in which chelae evolved from sub-chelae, we would also expect a more sideways-oriented fixed finger there.

### 4.7. Diversity of Grasping Structures in Kachin Amber

The new fossil finds add several new morphologies of grasping structures to Kachin amber. Truly chelate structures remain rare, including only a few decapodan crustaceans, especially crabs [38], but also tanaidacean crustaceans [39]. Among sub-chelate appendages, the diversity within the fauna is already relatively high, as demonstrated; for example, by Mantispidae [2]. Furthermore, grasping systems with two movable parts (see the introduction in [40]) have a high diversity in Kachin amber, in part outperforming the modern fauna [40]. This morphological diversity also indicates a high diversity of predatory strategies at that time; the Kachin amber forest was likely a habitat with a lot of threats, also highlighted by the plethora of co-occurring defensive structures [41].

## Data Availability

All data from this study are available in this paper and the associated papers.

## Supplementary Materials

The following supporting information can be downloaded at https://www.mdpi.com/article/10.3390/insects17040431/s1. Supplementary Figure S1: Amber piece with holotype of *Carcinonepa libererrantes* n. gen. n. sp. and its syninclusions, PED 4665, Kachin amber (Myanmar). (a). Overview of amber piece. (b). Rove beetle. (c). Pseudoscorpion. (d). Two springtails. (e–g). Mites. (h). Mite and beetle. (i). Springtail. (j,k). Mites. Supplementary Table S1: Information about the specimens included in the shape analysis and values of the effective principal components (PCs); the cited literature is listed in Supplementary Table S2. Supplementary Table S2: Literature references for the specimens included in the shape analysis, which are not cited in the main manuscript. Supplementary Files S1–S6: Detailed results of the shape analysis.
