# Supplementary figures and images for: A True Bug with a True but Unique Chela in 100 Million-Year-Old Amber
Source: Insects. 2026 Apr 17;17(4):431. doi: 10.3390/insects17040431 (PMC13116665; doi:10.3390/insects17040431)

-2S.D.

Mean

+2S.D.

PC1

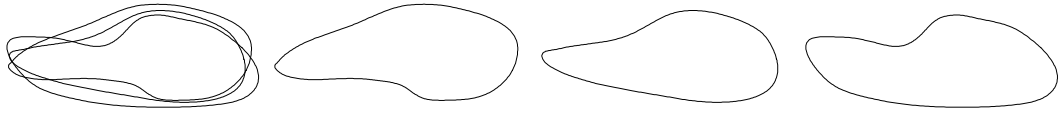

PC2

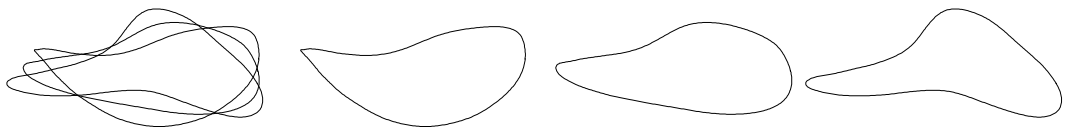

PC3

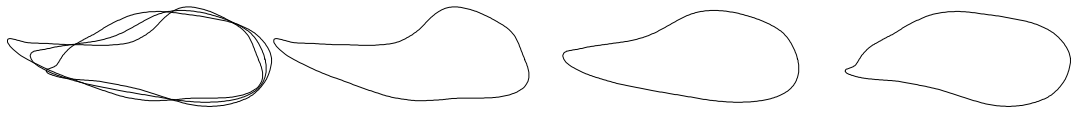

PC4

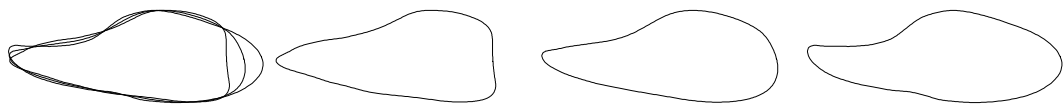

PC5

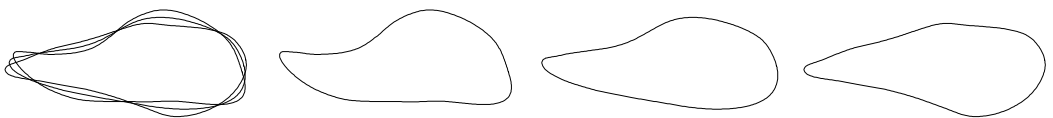

-2S.D.

Mean

+2S.D.

PC6

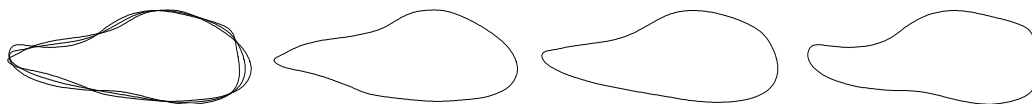

Supplement: Supplementary file 1 [file insects-17-00431-s001.zip › insects-4149200-supplementary/SupplFile1_Carcinonepa.pdf]

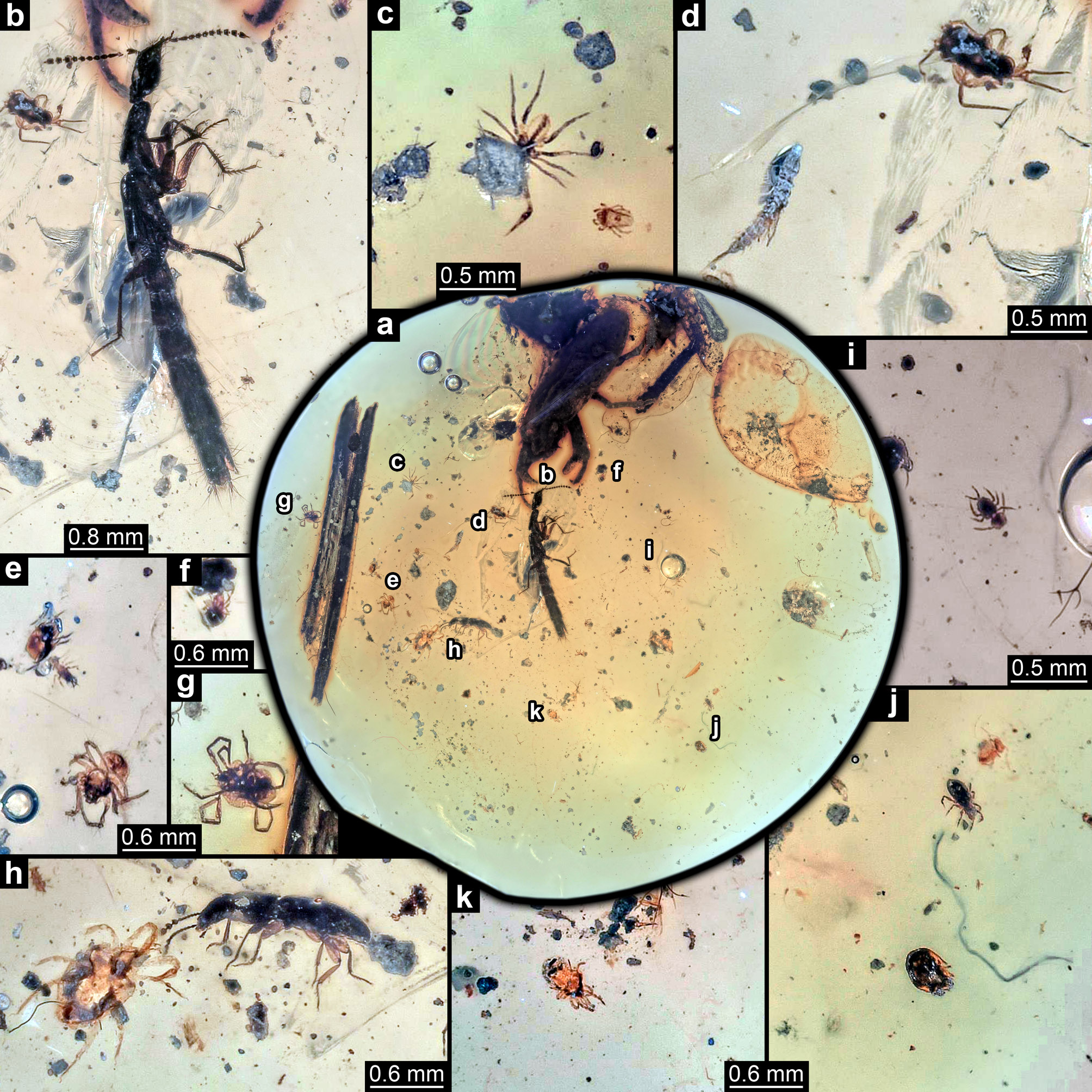

Supplement: Supplementary file 1 [file insects-17-00431-s001.zip › insects-4149200-supplementary/Suppl_Fig1_smCarcinonepa.jpg]
